# Reassortment Dynamics: Phylogeography and Evolution of H4N9 Influenza Viruses

**DOI:** 10.3390/pathogens14050469

**Published:** 2025-05-12

**Authors:** Nataliia A. Bobrova, Ekaterina D. Lisenenkova, Ekaterina S. Avsievich, Olga N. Mityaeva, Pavel Yu Volchkov, Andrey A. Deviatkin

**Affiliations:** 1 Federal Research Center for Innovator and Emerging Biomedical and Pharmaceutical Technologies, 125315 Moscow, Russia; lisenenkova_ed@academpharm.ru (E.D.L.); avsievich_es@academpharm.ru (E.S.A.); mityaeva_on@academpharm.ru (O.N.M.); volchkov_py@academpharm.ru (P.Y.V.); 2 Moscow Clinical Scientific Center N.A. A.S. Loginov, 111123 Moscow, Russia; 3 Laboratory of Postgenomic Technologies, Izmerov Research Institute of Occupational Health, 105275 Moscow, Russia

**Keywords:** virus, influenza A, reassortment, viral segment, H4N9

## Abstract

A characteristic feature of influenza A viruses is their high capacity for reassortment, significantly increasing their genetic diversity. This can lead to the formation of influenza A virus variants with unique phenotypic characteristics, particularly those with pandemic potential. Representatives of the H4N9 subtype are low-pathogenic influenza A (LPAI) viruses. Despite their low pandemic potential, these viruses may represent an important reservoir of genes for genetic exchange with other IAVs. Here, we analyzed the reassortment events of H4N9 viruses using all publicly available sequences. Several computational approaches, including phylogenetic reconstructions and reassortment detection algorithms (PDDM and PDCP), were used to identify phylogenetic incongruences. Numerous reassortment events were detected in H4N9 viruses, especially in the NS segment. This suggests extensive genetic exchange with other avian and mammalian IAVs. In addition, a comparison of phylogenetic and geographic patterns suggests that H4N9 viruses have undergone multiple trans-regional transmissions. These results suggest that LPAI viruses make a significant contribution to the overall influenza gene pool, increasing the likelihood of the emergence of new IAV variants with unpredictable phenotypic characteristics. However, our results suggest that the current understanding of the real distribution and genetic diversity is fragmented. Therefore, better monitoring and surveillance of H4N9 viruses should improve influenza pandemic preparedness.

## 1. Introduction

Influenza A viruses (IAVs) have a broad host range, with the greatest diversity found in wild bird species and established lineages circulating in poultry, pigs, humans, and other mammalian hosts [1]. The ability of a particular lineage to become infected is limited by species–specific barriers, but occasional spillover events can lead to continued transmission and initiate the spread of new lineages [1]. This process is referred to as zoonotic transmission. If the newly emerged IAV spreads efficiently from person to person, it can trigger a pandemic. IAVs are known to cause seasonal outbreaks and, occasionally, pandemics in humans [2]. The emergence of novel IAVs from animal reservoirs is of great concern as it can lead to serious public health consequences and have a negative impact on the economy. There have been numerous influenza pandemics throughout history. The best known are the Spanish flu in 1918, the Asian flu in 1957, the Hong Kong flu in 1968, and the H1N1 pandemic in 2009. These pandemics resulted in a significant number of cases, hospitalizations, deaths, and socio-economic disruption [3,4].

Changes in their genomes could give IAVs the ability to infect humans. The genomes of IAVs consist of eight single-stranded RNA segments (PB2, PB1, PA, HA, NP, NA, M, and NS) [5]. As segmented RNA viruses, IAVs can exchange gene segments by reassortment during co-infection. When two or more IAVs infect the same cell, a viral reassortment genome can be formed by combining the gene segments of the parent viruses [6]. It should be noted that reassortment is one of the main driving forces in the evolution of IAVs [7]. As a result of such a process, novel viruses with unpredictable phenotypes can emerge. For example, the 2009 influenza pandemic was caused by zoonotic transmission of the reassortant H1N1pdm09 virus from pigs to humans [8].

Non-structural (NS) protein is not incorporated into the virions, but is abundantly present in the nucleus of cells infected with the influenza virus at the beginning of the infection and also in the cytoplasm in later phases of the viral replication cycle. This NS protein inhibits the post-transcriptional processing of cellular antiviral pre-mRNAs [9]. The NS1 protein shows pleiotropic functions in the course of infection of mammalian cells. In addition, it plays a central role in the downregulation of the type I interferon response.

The taxonomy of IAVs is based on the subtyping of their HA and NA proteins. Nineteen HA subtypes and eleven NA subtypes are currently known [10]. Multiple combinations of different HA and NA subtypes lead to a large variety of IAVs. It should be noted that current knowledge of IAVs is focused on variants that pose a direct pandemic threat (e.g., H5N1, H5N8, H1N1, and H3N2) [8,11,12]. At the same time, it is known that other proteins of IAVs can also have an impact on their pathogenicity [13]. And other subtypes of IAVs, which at first glance do not pose an epidemic threat, may be involved in the emergence of new IAVs through reassortment. The main reservoir for IAVs is waterfowl [14]. In addition, IAVs can remain viable in freshwater for at least months [15,16]. Therefore, freshwater reservoirs along bird migration routes can be a melting pot where different IAVs can be conserved, leading to simultaneous infection of waterbirds with several different viruses. Such a situation makes reassortment possible.

The H4N9 virus has a broad host range and is frequently found in migratory birds such as teals, mallards, and ruddy turnstones [17]. The most important habitats of these birds are the USA, Canada, Japan, and China, where the H4N9 virus has been found. Of the European countries, specimens have so far only been found in Sweden. [17]. Although genetic analysis of H4N9 has shown that the viruses are low-pathogenic [18], the reassortment of this IAV type with H9N2, H7N3, and H11N9 has led to the emergence of the H7N9 subtype that caused the deadly avian influenza epidemic in China in 2013 [19]. Such examples highlight the importance of studying reassortments of IAVs. Wisedchanwet et al. [18] published a study in 2011 based on the analysis of 970 samples of Muscovy ducks among the bird species at a live bird market (LBM) in Bangkok, Thailand, in 2009. Among them, only 19 influenza-positive samples were detected, including subtypes H4N6 (n = 2), H4N9 (n = 1), and H10N3 (n = 16). In addition to subtyping, influenza A viruses can be classified into evolutionary lineages such as the Eurasian lineage or the North American lineage based on their host species and geographic region. A phylogenetic lineage refers to a group of viruses that share a common genetic origin for a particular gene. It is possible for a virus to have genes from different lineages, reflecting the different origins of the individual genes [20]. The results of the phylogenetic analysis showed that both H4N6 and H4N9 found in Thailand contained all gene segments derived from avian influenza A viruses of the Eurasian lineage. The pathogenicity of the representative of subtype H4N9, A/mallard/MN/263/99, has been demonstrated experimentally [21]. This virus, though genetically classified as low-pathogenic, caused a turkey’s death and development of moderate respiratory symptoms in a chicken, highlighting the need for cautious interpretation of genetic pathogenicity classifications.

H4N9 is not a frequently detected IAV subtype and is not one of the subtypes described as infectious to humans. Therefore, there is little or no information specifically on H4N9 reassortants. Reassortment with an H4N9 subtype is currently neither observed nor well studied. However, representatives of this subtype of the virus can lead to the death of birds. On the other hand, they can reassort with other subtypes, which can lead to the emergence of new variants of the influenza virus with unpredictable phenotypic characteristics. It should be noted that the first step in investigating the possibility of rapid change in this subtype may be to analyze the reassortment within the subtype. In this study, a complete genome sequence analysis of all known H4N9 viruses, including samples deposited at the National Center for Biotechnology Information (NCBI), was performed to investigate the phylogeography and evolution of the low-pathogenic H4N9 IAVs over a long period of time and at a global geographic scale.

## 2. Materials and Methods

### 2.1. Sequence Preprocessing

All available H4N9 nucleotide sequences were downloaded from the NCBI database. Sequences of viruses where not all segments were fully sequenced were removed. The sequences of the segments of viruses with complete genomes were concatenated with the Viral Segment Concatenator (VSC) [22] and the resulting sequences (n = 16) were aligned with MAFFT [23].

### 2.2. Recombination and Reassortment Analysis

Reassortment analysis was conducted using construction of the pairwise distance correspondence plot (PDCP) and pairwise distance deviation matrix (PDDM) [24]. For testing and comparing results from the general method, RDP5 [8] was used. SimPlot++ was used to make similarity plots [13].

Phylogenetic inference using the maximum likelihood (ML) method was performed with IQ-TREE [25]. The best-fitting model was automatically selected using the ModelFinder [25] implemented in the IQ-TREE package (v. 1.6.1) according to the Bayesian information criterion. The ultrafast bootstrap (BB) approximation (1000 replicates) was chosen to evaluate the statistical robustness of the internal branching order in the phylogeny. The tips of the phylogenetic tree were color-coded using the gradient_color.py script written in Python 3.12.2 [24].

## 3. Results

**Figure 1:**
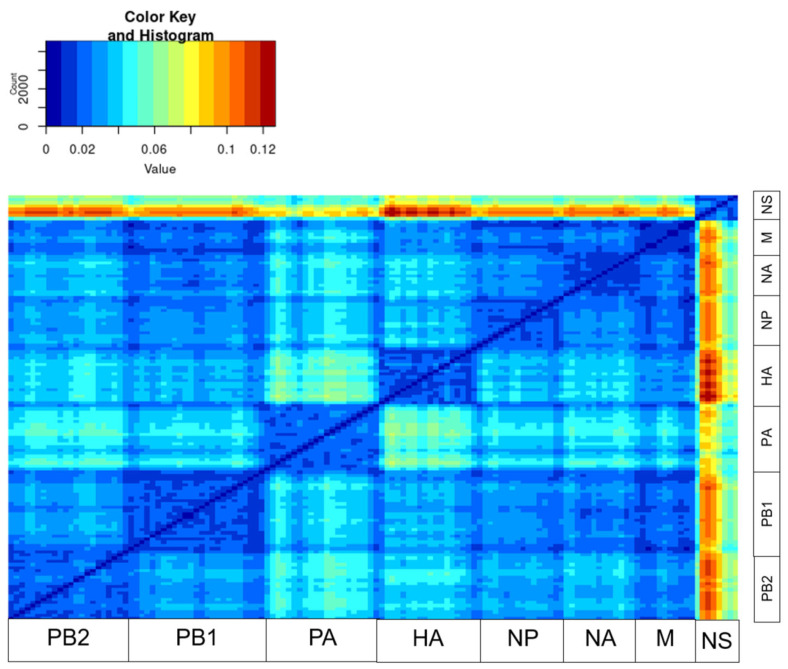
Pairwise distance divergence matrix (PDDM) for influenza viruses of subtype H4N9 (window = 200 nt, step = 100 bp). The color gradient scale shows the values of the root mean square error (RMSE) in the PDCP as a figure.

PDDM, using a sliding window (window = 200 nt, step = 100 bp) provided a comprehensive snapshot of the reassortment within the influenza A virus subtype H4N9. Figure 1 shows, in color, the levels of phylogenetic incongruence between the corresponding genomic regions. The red-colored pixels in Figure 1 indicate the most divergent genomic regions between which the phylogenetic incongruence was maximal. The most plausible explanation for this observation is a reassortment event. In contrast, the blue colors indicate low phylogenetic incongruence between the corresponding genomic regions. This visualization allows us to compare all phylogenetic relationships between all genomic regions of all sequences in the dataset simultaneously. According to this result (Figure 1), the maximum phylogenetic incongruence occurred between the eighth non-structural segment and all other segments.

**Figure 2:**
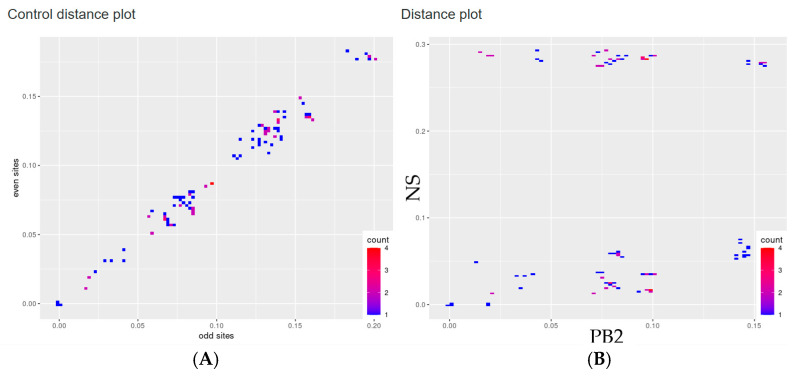
Pairwise nucleotide distance comparison plots (PDCPs) showing phylogenetic incongruence between selected genetic regions of H4N9 influenza viruses. Each dot represents a pair of raw nucleotide distances between two sequences in two genomic regions (axis labeling). The count value indicates the number of virus pairs per bin by a color gradient. The region of subsequent analysis was highlighted with the green boxes: (**A**) PDCP constructed for the concatenated genomic sequences of H4N9 viruses for which pairwise distances were calculated for even and odd sites (negative control) and (**B**) PDCP constructed for PB2 and NS segments of H4N9 influenza viruses.

To verify the divergence between the PB2 and NS segments, PDCPs were generated (Figure 2). The PDCPs were created for both genomic segments to illustrate the variance in evolutionary distances between them. Essentially, the PDCPs represent the proportion of different nucleotides between the PB2 and NS segments. The generation of pairwise distance comparison plots (PDCPs) entailed the division of all sequences into all possible pairs. The percentage of different nucleotides in the PB2 and NS genomic regions was then calculated for each pair of viruses. It is important to note that virus pairs showing varying levels of nucleotide divergence across distinct genomic regions are strong indicators of reassortment. The deviation from the main trend of the points in the PDCP diagram may indicate a different evolutionary history of the genomic regions studied. For example, A/mallard/Wisconsin/11OS3407/2011 (H4N9) and A/mallard/California/2584V/2011 (H4N9) had 98.6% identical nucleotides in the PB2 segment, but only 73.3% identical nucleotides in the NS segment. These drastic changes in the identity of the different segment sequences can most plausibly be explained by the presumed reassortments.

**Figure 3:**
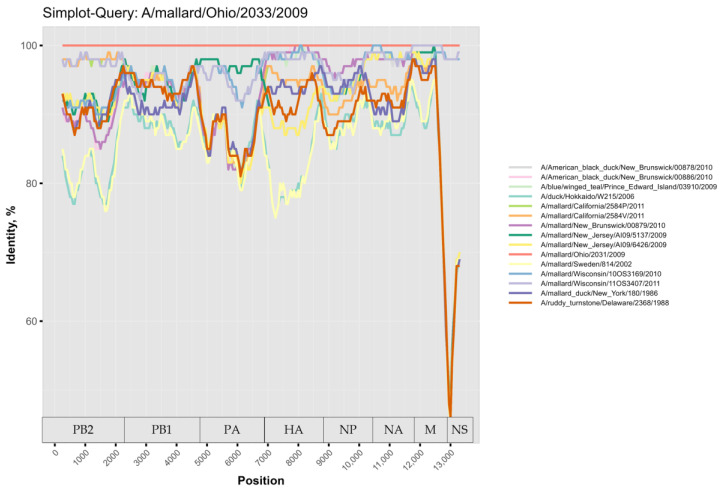
Similarity plot analysis (window = 500 nt, step = 50 bp) of the whole sequences.

For further investigation, the virus sequences were compared using a similarity plot. Several reassortments were detected, mainly in the NS segment, in agreement with PDDM and PDCP (Figure 3). In addition, the PB2 and HA segments also showed signs of a complex evolutionary history. For example, A/mallard/Ohio/2033/2009 (H4N9) (reference sequence in Figure 3) showed more than 90% identity with A/mallard/Wisconsin/11OS3407/2011 (H4N9) at the whole genome level. At the same time, A/mallard/Ohio/2033/2009 (H4N9) shared more than 90% of identical nucleotides with A/mallard/Wisconsin/11OS3407/2011 (H4N9) in the PB2, PB1, PA, NP, NA, and M segments, while these two viruses shared a drastically lower percentage of nucleotides in the HA and NS segments.

**Figure 4:**
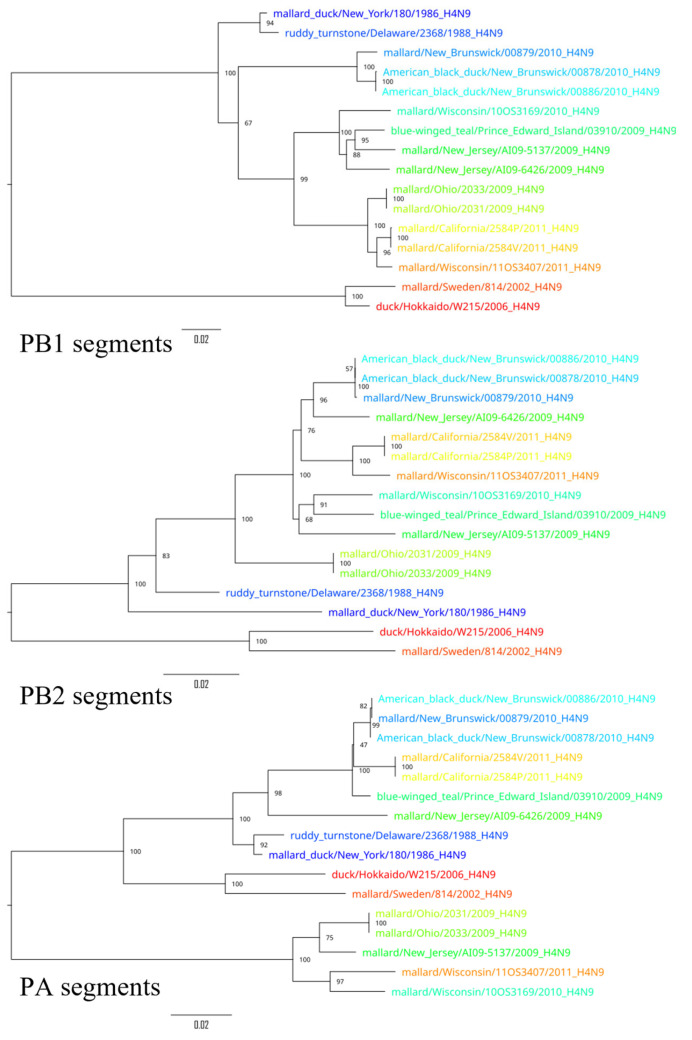
Phylogenetic trees for PB2, PB1, and PA segments. Each virus is color-coded to illustrate differences in the topology of the phylogenetic trees for the different segments.

**Figure 5:**
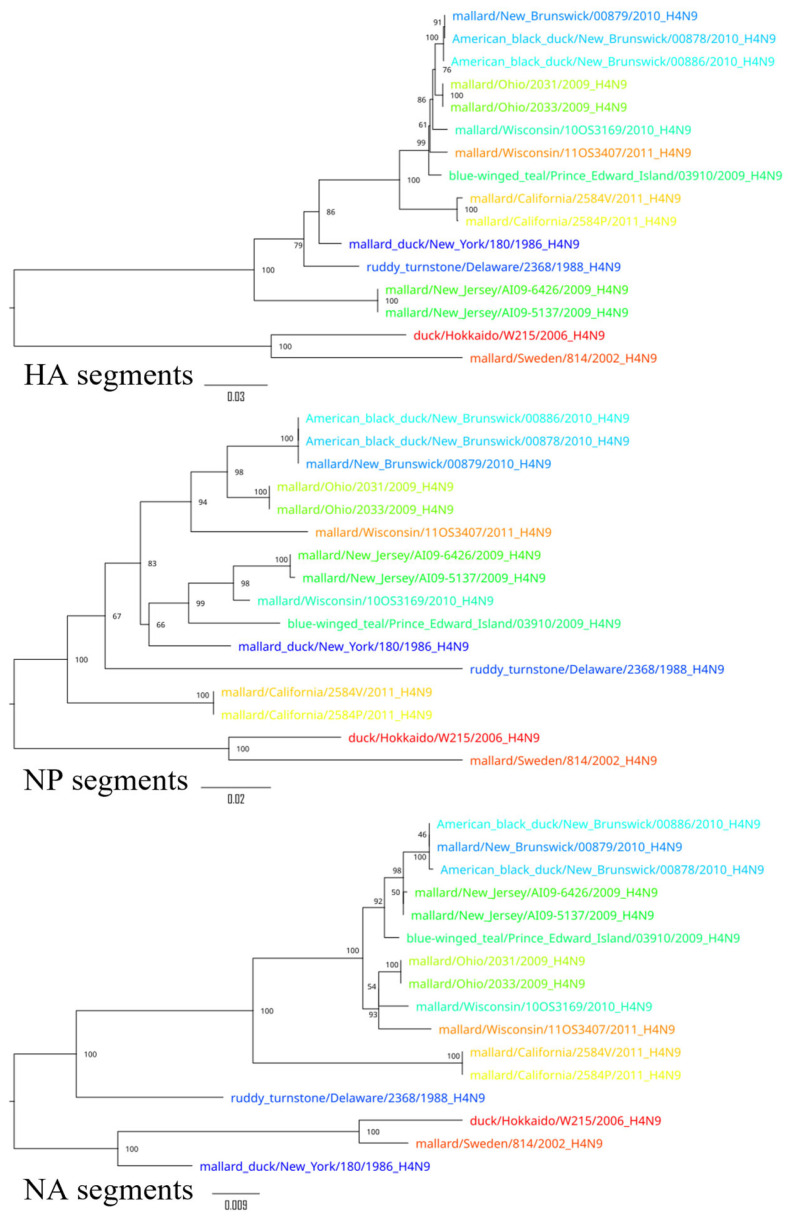
Phylogenetic trees for HA, NP, and NA segments. Each virus is color-coded to illustrate differences in the topology of the phylogenetic trees for the different segments.

**Figure 6:**
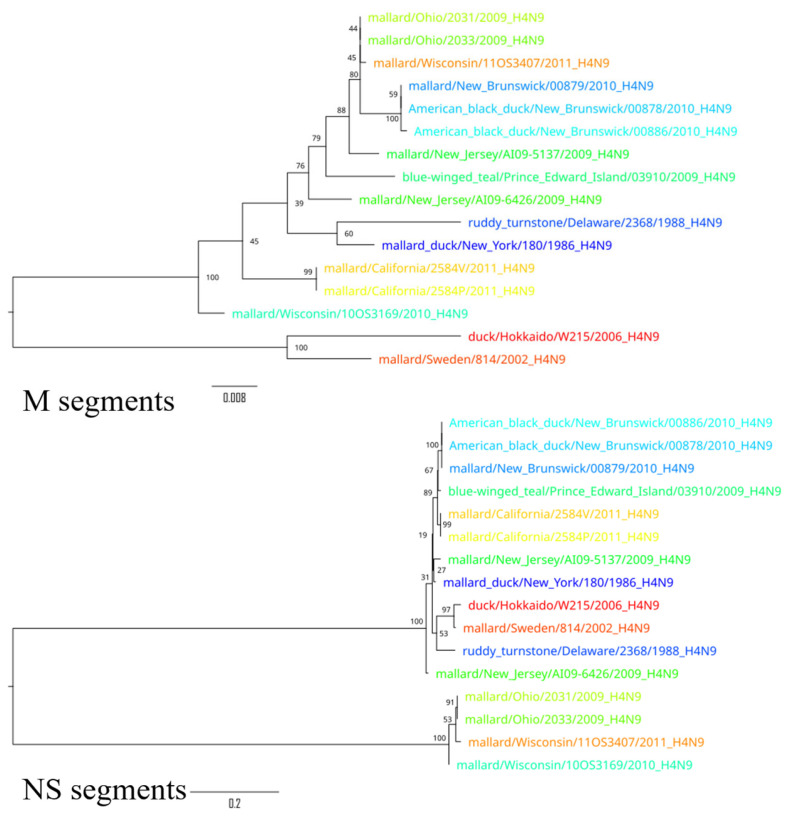
Phylogenetic trees for M and NS segments. Each virus is color-coded to illustrate differences in the topology of the phylogenetic trees for the different segments.

To demonstrate the results of the extensive reassortment within the H4N9 subtypes, phylogenetic trees were constructed for all segments (Figure 4, Figure 5 and Figure 6). To visualize the changes in the topology of the phylogenetic trees, each virus was assigned a specific color.

Phylogenetic trees constructed for different segments of the same viruses showed a different topology, suggesting possible reassortments. For example, A/duck/Hokkaido/W215/2006 and A/mallard/Sweden/814/2002 reliably formed an outgroup to other viruses based on the sequences of segments PB2, PB1, HA, NP, and M, while the phylogenetic relationship between these two viruses and other viruses was divergent according to other segment sequences. Moreover, the phylogenetic tree based on the sequences of the NS segments of the H4N9 subtype was drastically different from other phylogenetic trees—it formed two divergent clades whose viruses were only distantly related to each other. For example, the NS segments of A/duck/Hokkaido/W215/2006 and A/mallard/Ohio/2033/2009 differed in 26.7% (232 of 869) of the nucleotides. These differences in the topology of the phylogenetic trees constructed on the basis of the different segment sequences can be most plausibly explained by the frequent reassortment event that has determined the evolutionary history of the H4N9 subtype.

## 4. Discussion

Occasionally, zoonotic IAVs result in limited human-to-human transmission, but these are usually dead-end infections. In some cases, however, zoonotic IAV infections can cause pandemics [8]. In the last two decades, the incidence of zoonotic IAV infections has increased due to the endemicity of certain IAV lineages in agricultural species [13]. Genetic exchange through reassortment of segmented genomes often confers new genetic characteristics to IAVs that may affect the transmissibility and pathogenicity of the virus.

In recent decades, the approach to virus research, especially in the context of IAVs, has changed considerably. In the past, researchers focused primarily on viruses that posed a direct threat to humans, which corresponded to an anthropocentric paradigm. However, with the development of the One Health concept, it has become clear that human, animal, and ecosystem health are inextricably linked [26]. This paradigm shift is particularly evident when considering how low-pathogenic viruses circulating among wild and domestic birds can interact with highly pathogenic strains to create new viruses that are potentially harmful to humans. Pathogenicity is a phenotypic characteristic that determines the severity of the disease caused by the virus [27]. It should be noted that this phenotypic trait is predetermined by different proteins encoded in different segments of IAVs [28,29,30,31,32,33]. Thus, this polygenic trait depends on the combination of segments of IAV. However, during the reassortment process, new combinations of segments can arise, leading to potentially unpredictable phenotypic characteristics of the new virus. For example, between 1999 and 2001, an H7N1 avian influenza virus with low pathogenicity (an LPAI virus) mutated in northern Italy, leading to the emergence of a high pathogenicity (HPAI) strain. This virulent strain led to the death of over 16 million poultry and significant economic losses for the industry [34]. Phylogenetic analysis has revealed that the HPAI viruses are descended from low-pathogenicity viruses, and that they bear the greatest genetic similarity to a wild-bird isolate, A/teal/Taiwan/98. Further genetic analysis has identified specific deletions in the NA stalk region and the acquisition of additional glycosylation near the receptor-binding site of HA1 [35].

In 2009, a one-year active surveillance program for influenza A viruses in avian species was conducted at LBM in Bangkok, Thailand. Out of 970 samples collected, influenza A virus subtype H4N9 (n = 1) was isolated from a healthy Muscovy duck [5]. The results of the phylogenetic analysis showed that the H4N9 viruses found in Thailand contained all gene segments derived from influenza A viruses of the Eurasian lineage. Genetic analysis of the viruses also showed that the viruses had low pathogenic properties, with amino acids specific to avian influenza viruses. This could have been due to the specific, non-overlapping flight routes of wild and migratory birds between the Pacific and North American regions. The authors concluded that influenza A viruses of subtype H4N9 found in the live bird market could pose a potential risk to birds and humans [5].

In a study by Yamamoto N. et al., three samples were found to be positive for H4N9 during avian influenza surveillance in migratory waterfowl in Japan in 2006 [14]. In another study, active surveillance for influenza viruses was conducted from winter 2011 to spring 2012 in domestic ducks on farms along the migratory route in the Dongting Lake region of Hunan Province, China. In March 2012, three samples of the H4N9 subtype were detected among the samples [15].

**Figure 7:**
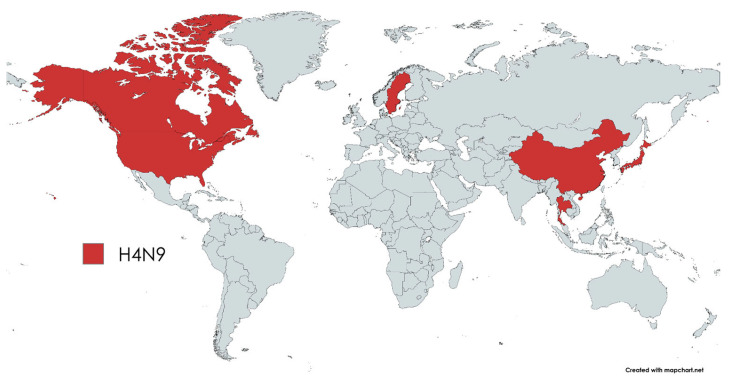
Detected AIV, subtype H4N9, by country. The map was created online at https://mapchart.net accessed on 7 March 2025.

The present analysis of H4N9 influenza viruses highlights a fundamental limitation in our current understanding of this virus lineage: our knowledge is fragmented. The detection of H4N9 in China, Japan, the USA, and Canada, but not in Russia, for example, (Figure 7) does not necessarily mean that the virus is not present in this region. Rather, it highlights the inadequacies of current surveillance measures and the fragmentation of current knowledge about the geographic distribution and genetic diversity of H4N9 IAVs. A thorough examination of the phylogenetic tree and geographical distribution shows that genetically similar viruses occur in geographically distant locations, for example, in Japan and Sweden. This observation suggests the possible existence of undiscovered intermediate strains or the facilitation of long-range viral movements by migratory birds. The fact that no H4N9 cases have been reported in certain areas may be due to inadequate sampling rather than the actual absence of the virus. This limitation is a recurring issue in avian influenza research, where surveillance biases can distort perceptions of virus distribution and evolution. The H4N9 IAV is a rarely detected virus that has been found in countries that do not border each other. At the same time, the actual situation in other regions of the world is unknown. It should be noted that IAV evolution is dynamic—the H7N1 outbreak in Italy [34] shows that a natural transition from LPAI to HPAI virus is possible.

The H4N9 subtype represents a rare reassortant combination within the avian influenza virus gene pool. According to the BLASTn algorithm, the HA of A/mallard/Minnesota/MN20-107E_T1/2020 (H4N9) shares more than 98% of identical nucleotides with the HA of A/mallard/Minnesota/MN20-197E_T1/2020 (H4N8) and A/blue-winged teal/Louisiana/UGAI18-1820/2018 (H4N6). In other words, the H4N9 viruses are closely related to those of the widespread H4N6 and H4N8 lineages. At the same time, the NA of A/mallard/Minnesota/MN20-107E_T1/2020 (H4N9) shares more than 98% of nucleotides with the NAs of the H11N9, H2N9, H5N9, H7N9, and H12N9 viruses. This indicates that the H4N9 subtype probably originated from a reassortment between the viruses of the above-mentioned subtypes. Such an event may have occurred in wild waterfowl, which serve as a natural reservoir for a variety of influenza A subtypes and often exhibit co-infections that facilitate genetic reassortment. The co-dispersal of the two parental subtypes in shared ecological niches creates the opportunity for reassortant genotypes such as H4N9 to evolve. These results emphasize the ongoing genetic plasticity of avian influenza viruses and the importance of continuous surveillance to detect new combinations with potential epidemiological significance.

The evolutionary background of these reassortment events is consistent with previous hypotheses that emphasize the persistence of antigenically distinct HA and NA lineages despite genetic sweeps in internal genes. As Worobey et al. suggested [36], a global sweep of internal genes likely occurred without eradicating the older diversity of HA and NA, reflecting immunologic selection for antigenic novelty in avian populations. This selective environment preserves multiple HA and NA subtypes and allows them to spread independently, even when internal genes are regularly homogenized. The estimated time to the most recent common ancestor (TMRCA) of the HA and NA subtypes is much longer than the time to the TMRCA of the internal gene segments.

The fact that the HA and NA subtypes are in sympatry with each other—as described by Dugan et al. [37]—suggests that each subtype occupies a fitness peak that is shaped by host immune pressure. This structure of the fitness landscape favors antigenic stability and limits the viability of intermediate or novel combinations unless they confer a distinct immunological advantage. In this context, the emergence and persistence of the H4N9 subtype could be a transient, but potentially important, event influenced by local immune landscapes and environmental factors. The primary function of the NS protein is the antagonization of IFN [38]. It can be hypothesized that the exchange of the NS protein between different viruses is a prerequisite for IAVs to evade the host’s protective mechanisms against viral infection. A number of experimental studies have demonstrated the efficacy of attenuated vaccines derived from alterations in the NS1 region in combating influenza A and B viruses in different host species [39]. High diversity in the NS region can therefore be considered an important factor in the adaptation of the virus to the host, and reassortment contributes to this diversity.

To improve global surveillance of avian influenza, particularly in underrepresented countries, it is critical to draw lessons from successful global health initiatives such as smallpox eradication [40]. These efforts have shown that, with coordinated international support, even regions with limited resources can effectively implement surveillance programs. To prevent the emergence of future pandemics in developed countries, it is essential that wealthier countries invest in building the necessary infrastructure for global surveillance of avian influenza. A robust, globally integrated surveillance system is crucial to track the spread of IAVs and mitigate risks in all regions as migratory birds cross political borders without control.

## 5. Conclusions

The study of evolution, especially the emergence of new strains through reassortment, is of great importance for public health. First, certain strains show a higher frequency of reassortment, suggesting that the risk of genetic exchange is not the same for all strains. Identifying the strains that reassort more frequently could increase pandemic preparedness or highlight potential targets for new treatments. This is particularly important if specific genetic markers can be identified that indicate increased genetic exchange, and thus contribute to better preparedness and targeted action. One example discussed in the literature is avian H9N2 viruses, which typically form the backbone for avian H7N9 viruses [16,17] and constitute a highly reassortant platform responsible for recent human infections [18,19]. Second, the replacement of a subset of segments, even at low abundance, could be important in the emergence of new strains under certain selection regimes, especially if these segments carry virulence or host tropism determinants [21].

Our results emphasize the patchiness of knowledge about highly reassortant and globally disseminated H4N9 IAVs. Although these viruses are considered low-pathogenic, they have the potential to serve as a genetic reservoir for the emergence of new, potentially high-risk strains. A global, standardized surveillance network is imperative to track such viruses more effectively.

## Data Availability

Raw sequence data used in the analysis, written in GenBank format, are available at https://github.com/AndreiDeviatkin/repo/blob/main/Reassortment_Dynamics%3A_Phylogeography_and_Evolution_of_H4N9_Influenza_Viruses/H4N9.gb (accessed on 8 May 2025).

## Abbreviations

The following abbreviations are used in this manuscript:

LPAI	Low-pathogenic influenza A
IAVs	Influenza A viruses
LBM	Live bird market
NCBI	National Center for Biotechnology Information
VSC	Viral Segment Concatenator
PDCP	Pairwise distance correspondence plot
PDDM	Pairwise distance deviation matrix
